# CEG: A joint model for causal commonsense events enhanced story ending generation

**DOI:** 10.1371/journal.pone.0286049

**Published:** 2023-05-23

**Authors:** Yushi Zhang, Yan Yang, Ming Gu, Feng Gao, Chengcai Chen, Liang He

**Affiliations:** 1 School of Computer Science and Technology, East China Normal University, Shanghai, China; 2 Shanghai Institute of AI for Education and School of Computer Science and Technology, Shanghai, China; 3 Xiaoi Robot Technology Co.,Ltd, Shanghai, China; The Catholic University of Korea, KOREA, REPUBLIC OF

## Abstract

With the success of pre-trained language models, the performance of story ending generation has been dramatically improved while remaining challenging due to the lack of commonsense reasoning ability. Most previous works mainly focus on using commonsense knowledge to enhance the implicit correlations between words but ignore the hidden causality of sentences or events. In this paper, we propose **C**ausal commonsense **E**nhanced joint model for story ending **G**eneration (CEG), which incorporates causal commonsense events knowledge to generate a reasonable story ending. Specifically, we first develop a commonsense events inference model trained on GLUCOSE, which converts static knowledge into a dynamic generation model to discover unseen knowledge. It uses prompts to produce various commonsense events behind the stories as pseudo-labels of the dataset. Then, we propose a joint model for the causal events inference task and the story ending generation task to inject inference knowledge into the generation, which consists of a shared encoder, an inference decoder, and a generation decoder. In the causal events inference task, we use the shared encoder and the inference decoder to reason the causal events behind each sentence of the story context to help the model better understand the story and provide long-distance dependencies for the story ending generation. In story ending generation, we combine the hidden states of the causal events with the story context to generate the story ending by the shared encoder and the generation decoder. We jointly train the model on two tasks so that the generation decoder produces the story endings that better match the clues. Experimental results on the ROCStories dataset show that our model outperforms the previous works, demonstrating the effectiveness of the joint model and the generated causal events.

## Introduction

Story ending generation aims to conclude a story and complete the plot given the context. It requires a model to understand the implicit commonsense knowledge beyond the text. Pre-trained language models such as GPT-2 [1] and Bert [2] have achieved great success in terms of fluency and informativeness. However, these models only focus on the surface meaning of the text, ignoring the commonsense knowledge behind the stories. Therefore, it is crucial to equip the model with commonsense reasoning ability to discover the commonsense knowledge underlying the story and further improve the performance of story ending generation.

**Fig 1:**
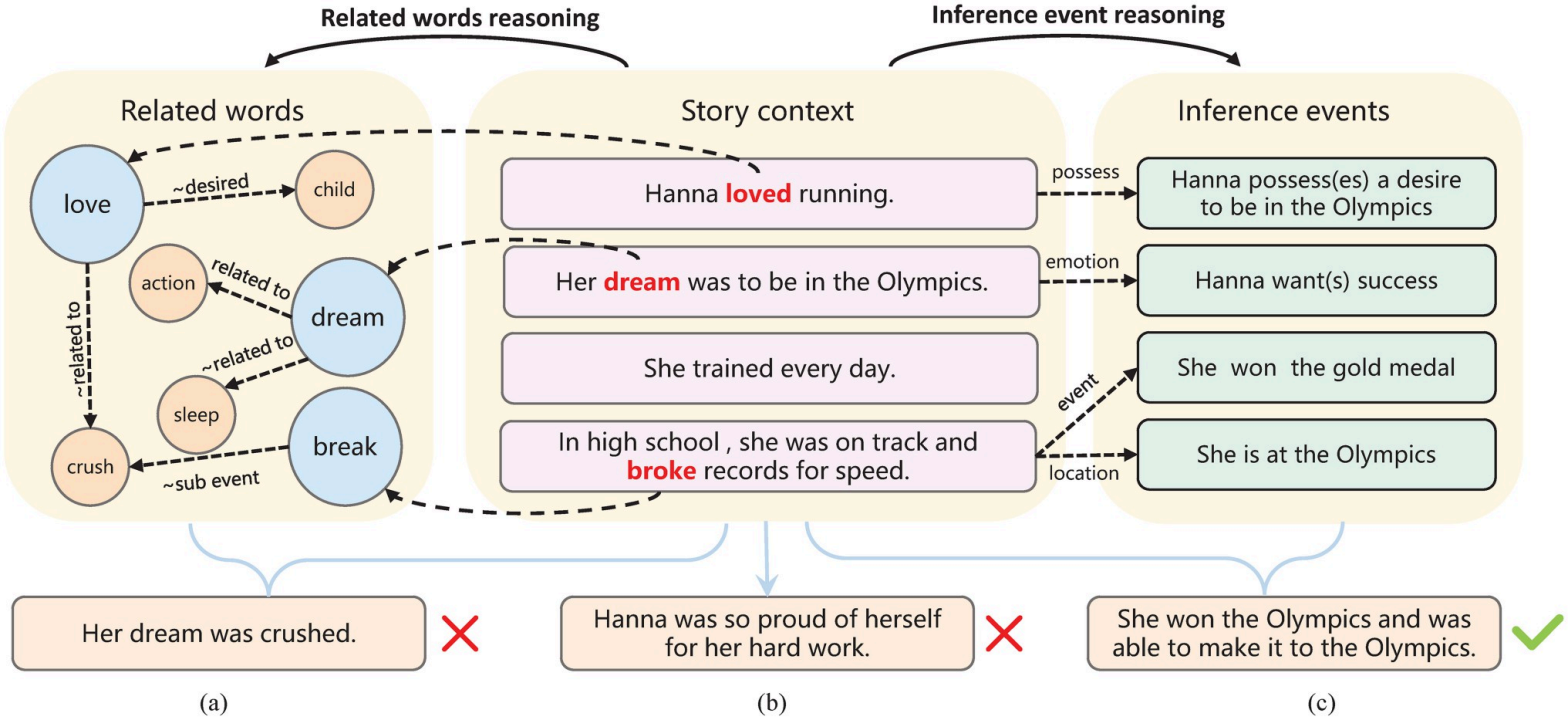
Story endings generated by different methods. Words in red are keywords of the story, which are linked to ConceptNet to infer related words (orange nodes) in method (a). Method (b) directly generates results according to the story context. Method (c) reasons the causal events behind each sentence by various relations on the commonsense events knowledge base and incorporates them into the ending generation.

Previous works mainly integrate ConceptNet [3] to enhance the commonsense reasoning ability. Specifically, some methods [4, 5] enrich word representations with their neighbors’ information on knowledge graphs while they neglect inference abilities. Others [6, 7] extract the subgraph from ConceptNet according to the keywords in the story and then explore the appropriate concepts for story generation. Although these studies have made great progress in commonsense reasoning, they purely focus on commonsense correlations at the word level, which might cause incoherence between endings and story clues [4–7]. As shown in Fig 1, the result of method (a) obtains relevant words of the keywords through the knowledge graph. The word “crush” is highly related to the keywords “love” and “dream” but leads to a wrong story ending which contradicts the story context.

In daily life, story development depends on the whole story rather than a single sentence or word, which implies that humans need to discover the logicality and causality among the story sentences and then capture the in-depth gist. Comparing the results of method (b) and method (c) in Fig 1, the former is directly generated by story context without reasoning, which lacks understanding of the whole story and only pays attention to the last two sentences. Meanwhile, the result of method (c) in Fig 1 shows that explicitly fusing the commonsense events behind the story context into the ending generation can help predict future events and generate a reasonable ending.

Luckily, some large-scale datasets of implicit event commonsense knowledge have been proposed, such as ATOMIC [8] and GLUCOSE [9]. Unlike ConceptNet, these knowledge bases focus on causal relations between events, which provide the basis for reasoning on the event level [8, 9]. Specifically, GLUCOSE [9] defines ten relations in daily life, including event, emotion, possession, etc. Therefore, with the help of commonsense knowledge about events, models can capture the underlying events in the context reasoned by different relations to understand the story better and make the ending generation coherent with the story clues.

In this paper, we propose **C**ausal commonsense **E**nhanced joint model for story ending **G**eneration (CEG), which infers multi-relational implicit commonsense events behind the context and integrates them explicitly into the story ending generation to make the ending more logical. Specifically, we propose a commonsense events inference model based on the BART [10] and fine-tune it on GLUCOSE [9]. It uses the prompts to discover underlying commonsense events of each story sentence by different relations as pseudo-labels. Furthermore, using the generation model can avoid being restricted to the size of the original knowledge base and discover unseen knowledge. Then, we devise a joint model for the causal events inference task and the story ending generation task, which consists of a shared encoder, an inference decoder, and a generation decoder. The causal events inference task uses the shared encoder and the inference decoder to reason underlying causal events of each story sentence, which assists model to understand the story from a global perspective and captures long-distance dependencies. In the story ending generation task, we encode the hidden states of causal events by the shared encoder and combine them with the story context to generate the story ending by the generation decoder. By sharing the parameter of the encoder, the model can better utilize its semantic information. Finally, we jointly train the model by the losses from two tasks to make them integrative and avoid error propagation. Experimental results on the ROCStories dataset [11] show that our model outperforms previous works, demonstrating the effectiveness of CEG and the generated commonsense events from multiple context sentences.

Our contributions are summarized as follows:

- We propose a story commonsense events inference model trained on GLUCOSE, which transforms static knowledge into a dynamic generative model. It infers commonsense events by prompts as pseudo-labels to train the joint model in the causal events inference task, which can improve the commonsense reasoning ability of the model.
- To the best of our knowledge, this is the first time that a joint model has been proposed for the causal events inference task and the story ending generation task, which infers causal events from each story sentence to provide long-distance commonsense information and improve the generation performance. The joint training of the model and the sharing of parameters make the inferred events more beneficial for the generation.
- We conduct experiments on the story ending generation dataset ROCStories, and the results demonstrate that our model generates more reasonable story endings than the previous works.

## Related works

Story ending generation is an important task for story generation, which aims to generate reasonable story ending according to the story context. However, it is difficult to capture commonsense knowledge only using surface information of the story. Therefore, some works constructed commonsense knowledge bases by exploring the relevance between the concepts or events and it is an effective way to integrate commonsense knowledge with generation models.

### Story generation

Story generation is the task of generating a reasonable story according to the leading context. It needs the model to generate multiple story sentences given the beginning of the story or some keywords [12–16]. [14, 16] utilized the VAE to encode the story plots or the keywords and then generated the follow-up stories. [15] introduced the event sequence as the trigger to help generate stories. In addition, some works [12, 13] used external knowledge to enhance story generation. [12] trained the model on ConceptNet and Atomic to obtain commonsense knowledge, which lacked inference according to the story. [13] utilized ConceptNet to reason the related words according to the keywords in the leading context but ignored the reasoning in the story context.

Different from story generation, story ending generation requires the model to generate a story ending according to the given story context. According to the result in [16], story plots become more and more complex and informative as the story progresses, so the story ending generation is more difficult than the story generation. [17] proposed a Seq2Seq model using adversarial training to enrich the diversity of the results. Compared to the maximum likelihood estimation, adversarial training prevented the model from generating a general ending for different story contexts. [18] combined the generation model with reinforcement learning to make the ending sensible. [19] drove the model to focus on the key phrases in the context to avoid the general ending. [20] turned the story context to the graph with relation dependency to improve the logical capability of the model. [21] proposed controllable story generation to generate the specific story ending for the given context and user’s intent. However, these models only trained on the large-scale corpus, which ignored commonsense knowledge.

### Commonsense knowledge

Commonsense knowledge bases play an essential role in enhancing the commonsense reasoning ability of models. ConceptNet [3] is a well-known knowledge graph connecting words and phrases with their relations. However, it mainly focuses on commonsense relevance between words and can not provide enough information to capture the relations between events. Unlike ConceptNet, ATOMIC [8] is composed of event sentences and their relations, such as “what impact does an event have on X”. [22] proposed COMET fine-tuned on ATOMIC to generate knowledge by a generative model. [9] proposed GLUCOSE, which included a story-specific statement paired with an inference rule generalized from the statement. The events of GLUCOSE are daily life stories that are more suited for the story ending generation task, so we use GLUCOSE as our commonsense knowledge base.

### Commonsense knowledge integrated generation

It has been proven that pre-trained language models like Bert [2] or GPT-2 [1] contain commonsense knowledge. However, these models still lack reasoning ability and can not obtain knowledge behind the context, which is impossible to solve only by increasing the model size [23]. So how to integrate commonsense knowledge into the generation model is a critical challenge in many tasks. [5, 6, 24] searched for the sub-graphs from the ConceptNet according to the context and integrated them into the model to enrich the commonsense knowledge of the model. However, these works only tried to infuse the word representation of commonsense knowledge into the model and ignored the reasoning. [7] made inferences on the ConceptNet to reason possible concepts for the generation but only focused on the word level, which ignored the relations of events. [25] extracted the representation from COMET to improve the commonsense reasoning of the model. [26] used a generation model fine-tuned on the commonsense knowledge base to complete the tasks.

Unlike these works, our model aims to infer implicit events behind the story to improve story ending generation. We first propose a commonsense events inference model fine-tuned on GLUCOSE, which transformed the static knowledge base into a dynamic generative model to infer the commonsense events behind the story as pseudo-labels. In addition, the generative model is more generalized than static knowledge and can generate some unseen knowledge. After that, we propose a joint model to generate the story ending through two tasks, causal events inference task, and story ending generation task. In the causal events inference task, we reason the causal events according to each sentence of the story by different relations, which provide global information and long-distance dependencies for the story ending generation. In the story ending generation task, we generate the ending by infusing the causal events with the story context. We jointly train the model and share the parameter of the encoder to make causal events more helpful to the story ending generation.

## Materials and methods

In this section, we will introduce our method in detail. To find out commonsense knowledge behind the story, we first propose commonsense events inference model finetuned on the GLUCOSE and discover implicit commonsense events as pseudo labels for the story. Then we design a joint model trained on the causal events inference task and story ending generation task. The causal event inference task reasons causal events by different relations and provides long-distance information for the story ending generation task. The joint training makes the causal events contain more information beneficial for the story ending generation and helps the model generate more reasonable story endings.

### Task formulation

In this paper, we exploit commonsense events behind the story context to help generate a more reasonable story ending. The task is formalized as follows: given the story context **X** = {*X*_1_, *X*_2_, ⋯, *X*_*m*_}, where *m* is the sentence number of context, we need to generate a reasonable ending *Y* and complete the plot. Each sentence *X*_*i*_ contains several words $X_{i}=\left\{x_{1}^{\left(i\right)},x_{2}^{\left(i\right)},\cdots ,x_{l_{i}}^{\left(i\right)}\right\}$. To perceive the logicality behind the story, we first reason the causal events **C** = {*C*_11_, *C*_12_, ⋯, *C*_1*n*_, *C*_21_, ⋯, *C*_*mn*_} behind the story through relation set *R* = {*r*_1_, *r*_2_, ⋯, *r*_*n*_}, where *n* is the number of different relation types and *C*_*ij*_ is the causal event inferred by story sentence *X*_*i*_ and relation *r*_*j*_. Then we combine causal events with story context to generate a reasonable story ending *Y*, formally as:

$$\begin{array}{c} Y=argmax_{Y}\;P\left(Y|X,C\right) \end{array}$$ (1)

### Commonsense events inference

To generate a reasonable story ending, the model is required to understand the causal relevance between different events of the story. We use the external commonsense knowledge base GLUCOSE [9] to train the model and assist the model in inferring the events from the story context. Since GLUCOSE cannot cover all events in the story and link them correctly to the corresponding event in the knowledge base, we design a commonsense events inference model fine-tuned on GLUCOSE. It can transform static knowledge into a dynamic generative model and generate commonsense events unseen in the knowledge base. And then, we employ it to reason about the commonsense events of the story as pseudo-labels to augment the data and train the joint model.

We fine-tune a BART [10] model on GLUCOSE. At the encoding step, we input the entire story context **X** = {*X*_1_, *X*_2_, ⋯, *X*_*m*_} with story ending *Y* as input to the BART encoder and get their representation *H*. At the decoding step, we use the prompts of different relations to guide the model generation. For each relation in GLUCOSE, we set a prompt like “emotion that motivates 〈sentence〉 is”, where the special token 〈sentence〉 is replaced by the specific sentence *X*_*i*_ needing inference. More detailed relation categories and prompts are shown in Table 1. Together with the representation from the encoder, the prompt *Prompt*_*ij*_ of the relation *r*_*j*_ and the specific sentence *X*_*i*_ will be fed into the BART decoder to generate the corresponding event *I*_*ij*_.

$$\begin{array}{cc} H & =BARTEncoder(X,Y) \end{array}$$ (2)

$$\begin{array}{cc} I_{ij} & =BARTDecoder(H,Prompt_{ij}) \end{array}$$ (3)

At the inference step, we use the same format as the training step to generate commonsense events **I** = {*I*_11_, *I*_12_, ⋯, *I*_1*n*_, *I*_21_, ⋯, *I*_*mn*_} for each story, where *I*_*ij*_ is the commonsense event inferred from story sentence *X*_*i*_ and relation *r*_*j*_. These commonsense events are regarded as pseudo-labels to train the model in the causal events inference task.

**Table 1:** Relations defined in GLUCOSE.

Category	Relation Description	Prompt
**Cause**	Event that directly causes or enables 〈sentence〉	event that directly causes 〈sentence〉is
	Emotion or basic human drive that motivates 〈sentence〉	emotion that motivates 〈sentence〉is
	Location state that enables 〈sentence〉	location state that enables 〈sentence〉is
	Possession state that enables 〈sentence〉	possess state that enables 〈sentence〉is
	Other attributes enabling 〈sentence〉	other attributes that enables 〈sentence〉is
**Effect**	Event that 〈sentence〉directly caused or enabled	event that 〈sentence〉directly cause is
	An emotion that is caused by 〈sentence〉	emotion that caused by 〈sentence〉is
	A change in location that 〈sentence〉results in	change in location that 〈sentence〉results in is
	A change of possession that 〈sentence〉results in	change of possession that 〈sentence〉results in is
	Other changes in property that 〈sentence〉results in	other changes that 〈sentence〉results in is

The relations are divided into five types: event, emotion, location state, possession state, and other attributes, which describe the causes and effects between sentences. We set a prompt for each relation to guide the generation of the model.

### Causal commonsense enhanced joint model for story ending generation

**Fig 2:**
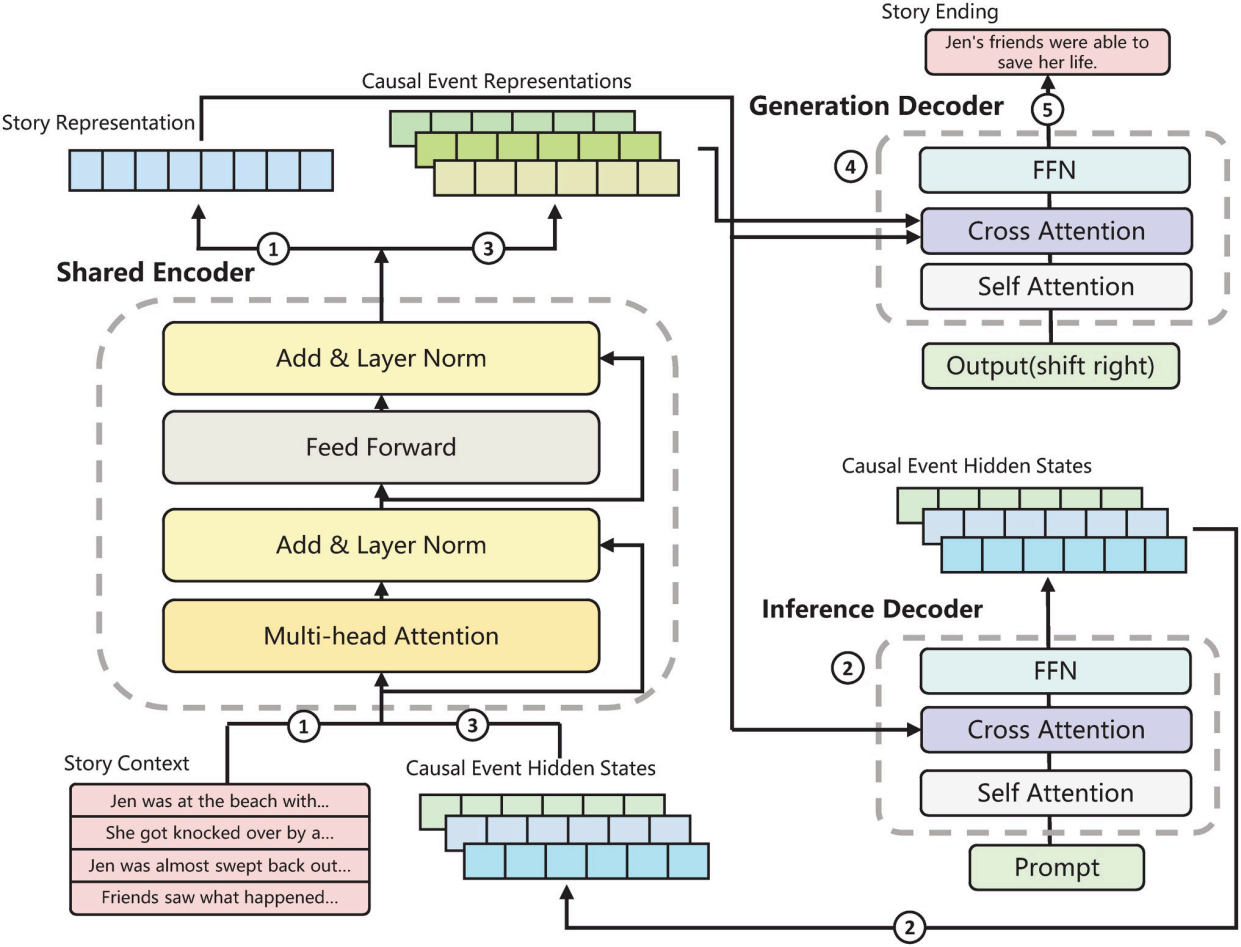
The architecture of CEG. The model architecture consists of three components, a shared encoder, an inference decoder, and a generation decoder. The causal events inference task uses the shared encoder and the inference decoder to generate causal events for the story context. The story ending generation task applies the shared encoder and the generation decoder, which integrates the causal events and the story context to finish the ending.

We propose **C**ausal commonsense **E**nhanced joint model for story ending **G**eneration (CEG) to infuse commonsense knowledge into story ending generation. Fig 2 illustrates the model architecture, which consists of a shared encoder, an inference decoder, and a generation decoder. The model is jointly trained on the causal events inference task and the story ending generation task. The causal events inference task aims to generate causal events relevant to the story context using the shared encoder and inference decoder. Each causal event is inferred by the corresponding sentence in the story context using a specific causal relation. Then we generate the story ending according to the story context and the hidden states of causal events in the story ending generation task by the shared encoder and the generation decoder. Two tasks share the same encoder to better utilize its linguistic properties. At last, we train the model by the losses from two tasks jointly to make the causal events more adaptive for story ending generation.

#### Causal events inference

The commonsense knowledge behind the story is implicit, and the language model usually can not capture them just by the story context. So we use external commonsense knowledge to train the model reasoning events related to the story. The causal events inference uses an encoder-decoder architecture model [27], which consists of a shared encoder and an inference decoder. We concatenate the sentences in the story context **X** = {*X*_1_, *X*_2_, ⋯, *X*_*m*_} with special tokens, which can be denoted as *X*_*concat*_ = [*X*_1_, 〈/*s*〉, *X*_2_, 〈/*s*〉, ⋯, 〈/*s*〉, *X*_*m*−1_], and then input it into the shared encoder to get the story representation *H*_*s*_:

$$\begin{array}{cc} H_{s} & =SharedEncoder(X_{concat}) \end{array}$$ (4)

$$\begin{array}{cc} & =FFN(MultiHead\left(X_{concat},X_{concat},X_{concat}\right)) \end{array}$$ (5)

where the shared encoder is a Transformer-based [28] encoder. FFN is a fully connected feed-forward network containing two linear transformations with a ReLU activation. MultiHead is the multi-head attention layer of the Transformer, which encodes the value *V* according to the attention weights calculated by query *Q* to key *K*.

$$\begin{array}{cc} MultiHead(Q,K,V) & =Concat(head_{1},\cdots ,head_{h}) \end{array}$$ (6)

$$\begin{array}{cc} where\;head_{i} & =Attention(QW_{i}^{Q},KW_{i}^{K},VW_{i}^{V}) \end{array}$$ (7)

$$\begin{array}{cc} Attention(Q,K,V) & =softmax(\frac{QK^{T}+M}{\sqrt{d_{k}}})V \end{array}$$ (8)

where $W_{i}^{Q},W_{i}^{K}$ and $W_{i}^{V}$ are the parameters of the attention mechanism, *M* is the attention mask, and *d*_*k*_ is the dimension of the query and key.

**Fig 3:**
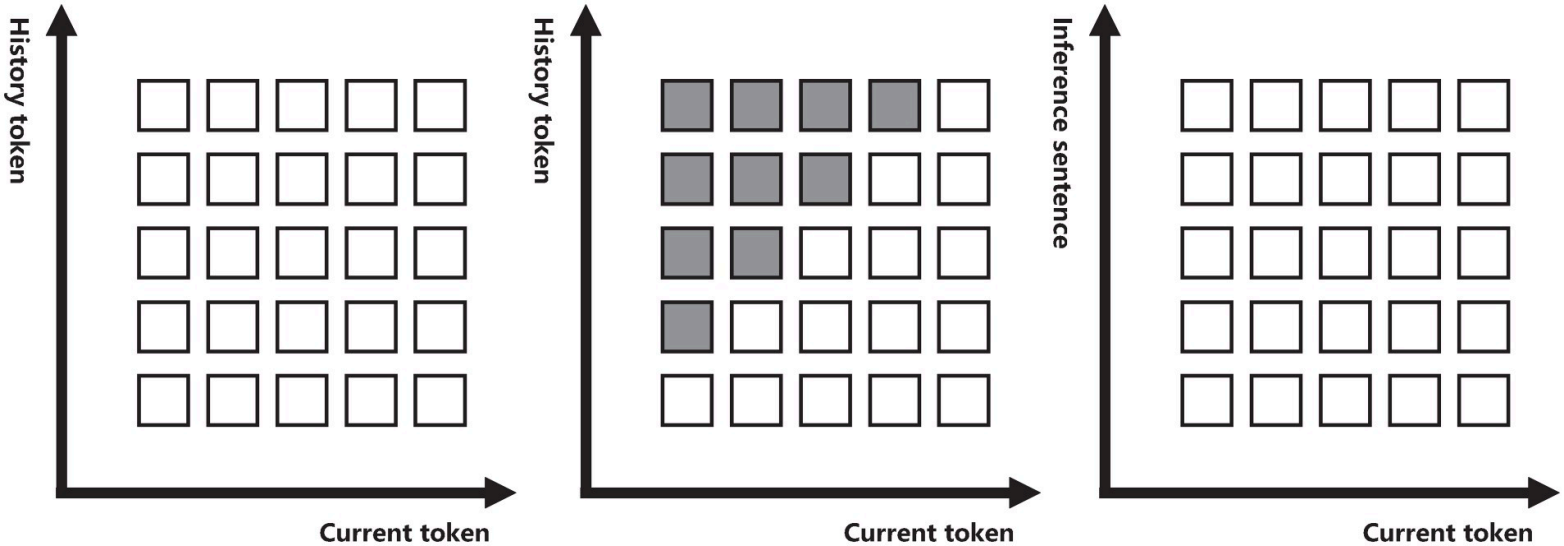
Mask of different modules. The first mask is the encoder mask, which is bidirectional, and each token accesses the information of other tokens. The second mask is the mask of the self attention in the decoder, which is a left-to-right mask, and each token only accesses information of history tokens. The third mask is the mask of the cross attention in the decoder, where each token access all information from the encoder.

At the decoding step, we use the inference decoder to generate the causal events **C** = {*C*_11_, *C*_12_, ⋯, *C*_1*n*_, *C*_21_, ⋯, *C*_*mn*_} by prompts of the relation set *R* and story representation *H*_*s*_, where *m* is the sentence number of context and *n* is the number of relations. The inference decoder is a Transformer-based decoder consisting of self attention, cross attention, and a fully connected feed-forward network. We input the prompts shown in Table 1 into the decoder and guide the model to generate causal events. The *Prompt*_*ij*_ is the prompt of story sentence *X*_*i*_ and relation *r*_*j*_, which is used to infer the causal event *C*_*ij*_. The story representation *H*_*s*_ is input into the cross attention and calculated the attention weight with the hidden states from the self attention. Finally, we get the hidden states of the causal events **O** = {*O*_11_, ⋯, *O*_1*n*_, ⋯, *O*_*mn*_} and their corresponding sentences **C** = {*C*_11_, ⋯, *C*_21_, ⋯, *C*_*mn*_}:

$$\begin{array}{cc} C_{ij} & =InferenceDecoder(H_{s},Prompt_{ij}) \end{array}$$ (9)

$$\begin{array}{cc} o_{self}^{\left(k\right)} & =MultiHead(x^{\left(k\right)},x^{\left(1:k-1\right)},x^{\left(1:k-1\right)}) \end{array}$$ (10)

$$\begin{array}{cc} o_{cross}^{\left(k\right)} & =MultiHead(o_{self}^{\left(k\right)},H_{s},H_{s}) \end{array}$$ (11)

$$\begin{array}{cc} o^{\left(k\right)} & =FFN(o_{cross}^{\left(k\right)}) \end{array}$$ (12)

$$\begin{array}{cc} {\hat{y}}^{\left(k\right)} & =LMHead(o^{\left(k\right)}) \end{array}$$ (13)

where $o_{self}^{\left(k\right)}$ is the output of the self attention in step *k*, and $o_{cross}^{\left(k\right)}$ is the output of the cross attention. In self attention, each token *x*^(*k*)^ calculates attention weight with history tokens *x*^(1 : *k*−1)^, and the self-attention mask is the left-to-right form to ensure each token only depends on the history tokens, as shown in Fig 3. At the cross attention, we calculate attention weight between the output hidden states of self attention $o_{self}^{\left(i\right)}$ and story representation *H*_*s*_. Finally, we get the hidden state *o*^(*k*)^ and its corresponding token ${\hat{y}}^{\left(k\right)}$, where *o*^(*k*)^ is the *k*th hidden state of *O*_*ij*_, ${\hat{y}}^{\left(k\right)}$ is the *k*th token of the *C*_*ij*_.

We calculate the loss of the casual events $L_{c}$ between generated results **C** and the pseudo-labels **I**. The loss function is the negative log-likelihood function:

$$\begin{array}{c} L_{c}=-\sum_{i=1}^{m}\sum_{j=1}^{n}\sum_{k=1}^{l_{ij}}P\left(y^{\left(k\right)}\right)log\left(P\left({\hat{y}}^{\left(k\right)}\right)\right) \end{array}$$ (14)

where *m* is the number of sentences in story, *n* is the number of inference events, and *l*_*ij*_ is the sequence length of the causal event *C*_*ij*_, *y*_*k*_ is the *k*th token of *I*_*ij*_ and ${\hat{y}}^{\left(k\right)}$ is the *k*the token of *C*_*ij*_. *P*(⋅) represents the probability score from the model output. *log*(⋅) is the base-2 logarithm.

Furthermore, we use the hidden states of the causal events **O** = {*O*_11_, ⋯, *O*_1*n*_, ⋯, *O*_*mn*_} in the story generation task instead of the causal events sentence **C**, so the loss from the story ending generation task can optimize the generation of the causal events and make causal events more precisely.

#### Story ending generation

Starting with the hidden states of the causal events **O**, we apply a Transformer-based encoder-decoder architecture model with the shared encoder and the generation decoder to generate a story ending. To utilize its linguistic and semantic information, we share the same encoder in the causal events inference task. First, we use the shared encoder to encode the hidden states **O** into the intermediate representations $H_{c}=\left\{H_{c_{11}},\cdots ,H_{c_{1n}},\cdots ,H_{c_{mn}}\right\}$. Each hidden state *O*_*ij*_ encode into $H_{c_{ij}}$ individually. We concatenate each intermediate representation $H_{c_{ij}}$ and story representation *H*_*s*_, then we input concatenation into the generation decoder to yield the result, which is applied to compute the attention weight with each step’s hidden states in the cross attention of the generation decoder.

$$\begin{array}{cc} H_{c_{ij}} & =SharedEncoder(O_{ij}) \end{array}$$ (15)

$$\begin{array}{cc} {\hat{y}}^{\left(k\right)} & =GenerationDecoder(\left[H_{s};H_{c_{11}};H_{c_{12}};\cdots \right],{\hat{y}}^{\left(1:k-1\right)}) \end{array}$$ (16)

where $\hat{Y}=\left\{{\hat{y}}^{\left(1\right)},{\hat{y}}^{\left(2\right)},\cdots ,{\hat{y}}^{\left(l\right)}\right\}$ is the generated story ending of the model. ${\hat{y}}^{\left(k\right)}$ is the *k*th token of the ending, and ${\hat{y}}^{\left(1:k-1\right)}$ are history tokens.

The story ending loss is a negative log-likelihood between generated ending $\hat{Y}$ and the golden ending *Y*.

$$\begin{array}{c} L_{s}=-\sum_{k=1}^{l}P\left(y^{\left(k\right)}\right)log\left(P\left({\hat{y}}^{\left(k\right)}\right)\right) \end{array}$$ (17)

where ${\hat{y}}^{\left(k\right)}$ is the *k*th token of generated ending $\hat{Y}$ and *y*^(*k*)^ is the *k*th token of the golden ending *Y*. *P*(⋅) represents the probability score from the model output and *log*(⋅) is the base-2 logarithm.

#### Training objective

The training objective is the sum of the losses in two tasks:

$$\begin{array}{c} L=L_{c}+L_{s} \end{array}$$ (18)

where $L_{c}$ is the loss of the causal events inference task and $L_{s}$ is the loss of the story ending generation task. And then, we can train the model jointly on two tasks by the total loss L.

## Results

To evaluate the ability of our model, we conduct experiments on ROCStories [11] and compare our model with other baseline models. We both use automatic metrics and human evaluation to measure the performance of the models. Then, we explore the effectiveness of different parts in our model and represent some cases of models to analyze the difference between them.

### Dataset

We conduct experiments on the ROCStories dataset, which contains stories describing daily life, and each story consists of five sentences. The task of the dataset is to use the first four sentences as story context to generate a reasonable story ending. We use the data splits in [7] as Table 2 shown. To highlight causal events behind the stories, we employ GLUCOSE as our commonsense knowledge base, containing the commonsense events inferred from the story context. The size of GLUCOSE is shown in Table 2.

**Table 2:** Data split.

ROCStories			GLUCOSE		
Train	Dev	Test	Story num	Train	Dev
89993	4080	4080	4881	169185	5068

### Implementation details

Five types of relations defined in GLUCOSE cover the causes and effects of the specific sentence, including event, emotion, location, possession, and other attributes, as shown in Table 1. For each story sentence and relation, we infer one causal event. We use the base version of BART parameters to initialize the shared encoder and two decoders. Both the encoder and decoder have 768-dimensional hidden states, 6 layers, and 12 attention heads. All our experiments are conducted on a cloud computing instance with Intel Xeon Gold 6130 CPU and Nvidia Tesla A100 40GB GPU.

### Automatic metrics

We adopt following automatic metrics to evaluate the performance of models.

**BLEU-n:** BLEU-n measures the similarity between generated endings and ground truths. It calculate the n-gram overlap against the golden story ending *Y* and the generated story ending $\hat{Y}$. The higher BLEU score, the closer the story ending generated by the model is to the correct answer, and the better the model’s generation performance.

$$\begin{array}{c} BLEU-n=\frac{\sum_{n-gram\in Y}Count_{match}\left(n-gram\right)}{\sum_{n-gram\in \hat{Y}}Count\left(n-gram\right)} \end{array}$$ (19)

where *Count*_*match*_(*n* − *gram*) is the number of the n-gram that both appeared in generated story ending $\hat{Y}$ and the golden story ending *Y*. *Count*(*n* − *gram*) is the number of n-gram in the golden story ending *Y*. In our experiment, we evaluate the results with *n* = 1, 2, 3, 4, which are represented as BLEU-1, BLEU-2, BLEU-3, and BLEU-4.

**DISTINCT-n:** DISTINCT-n measures the diversity of the generated story endings. It calculates the ratio of the distinct n-gram in the entire generated story endings. The higher DISTINCT score, the more diverse the results generated by the model are.

$$\begin{array}{c} DISTINCT-n=\frac{Count_{distinct}\left(n-gram\right)}{Count(n-gram)} \end{array}$$ (20)

where *Count*_*distinct*_(*n* − *gram*) is the number of the distinct n-gram in entire generated story endings. *Count*(*n* − *gram*) is the number of n-gram in entire generated story endings. In our experiment, we evaluate the results with *n* = 1, 2, 3, 4, which are represented as DISTINCT-1, DISTINCT-2, DISTINCT-3, DISTINCT-4.

In the test step, we use the checkpoint with the best BLEU-1 on the development set as the final model to generate endings on the test set. In addition, we also conduct a human evaluation to investigate the model’s ability.

### Baselines

We compare our model with several promising baselines:

**Seq2Seq** [7]: A simple encoder-decoder model based on long short-term memory (LSTM) with an attention mechanism.

**IE+GA** [5]: IE+GA uses an incremental encoder to model the story context from different sentences. After that, a multi-source attention mechanism is applied to gather the information from the story and the ConceptNet.

**T-CVAE** [16]: A conditional variable autoencoder based on Transformer for story ending generation, which uses a latent variable to learn the distribution of story.

**GPT2** [1]: We finetune a GPT2 model on the ROCStories dataset to generate the story ending.

**GRF** [7]: A generation model enables pre-trained models with dynamic multi-hop reasoning on the external commonsense knowledge graph to generate the result.

### Result

The results of automatic metrics are summarized in Table 3.

**Table 3:** The results on the ROCStories.

Models	BLEU-1	BLEU-2	BLEU-3	BLEU-4	DISTINCT-1	DISTINCT-2	DISTINCT-3	DISTINCT-4
Seq2seq [7]	19.100	5.500	-	-	-	18.100	36.000	-
IE+GA [5]	20.800	6.400	-	-	-	14.000	28.000	-
T-CVAE [16]	25.700	9.900	4.800	3.100	3.100	18.900	-	-
GPT-2 [1]	25.075	9.885	5.178	3.027	7.891	29.620	49.978	64.642
GRF [7]	26.090	10.954	5.980	3.601	**9.016**	**37.796**	62.213	76.946
CEG (ours)	**26.886**	**11.712**	**6.585**	**4.110**	7.035	36.190	**65.225**	**82.114**

The numbers listed in the table are the percentages of BLEU or DISTINCT.—indicates the original paper didn’t report the result on the respective metric. The higher BLEU and DISTINCT scores, the better performance of the model.

According to Table 3, we can observe the following:

- The results of our model show an improvement over the baseline models on diverse metrics. It indicates that the commonsense knowledge of events contributes to the story ending generation. The model obtains the commonsense reasoning ability through joint training and infers causal commonsense events to improve the story ending generation. It allows the generation model to understand the implicit commonsense knowledge behind the story and generate more reasonable endings.
- Comparing the results of CEG and GRF, our model is higher than GRF on BLEU-1 to BLEU-4 by 0.8, 0.7, 0.6, and 0.5, which indicates that commonsense knowledge is more helpful to the story ending generation. Causal events of the story context can predicate the story’s development and lead to a logical ending. In addition, causal events help the CEG perform better on sentence diversity and get higher DISTINCT-3 and DISTINCT-4 by 3.0 and 5.1 respectively. At the same time, GRF searches the related words on ConceptNet to enhance the story context DISTINCT-1 and DISTINCT-2 by 2.0 and 1.6, which leads to better lexical diversity.
- We find that the pre-trained language models improve generated results on language diversity by comparing the results between pre-trained language models (GPT-2, GRF, CEG) and traditional language models (Seq2Seq, IE+GA, T-CVAE). The DISTINCT-1 and DISTINCT-2 scores of GPT-2 were higher than the T-CVAE by 4.8 and 10.7. However, the pre-trained model only uses surface semantic information and lacks reasoning ability. It generates the story ending through the related information in the dataset, which may cause errors in unseen scenarios. To avoid these problems, we incorporate commonsense knowledge into the model to enhance the reasoning ability, which can improve our model on BLEU-1 by 1.8 compared with GPT-2.

### Human evaluation

We conduct the human evaluation for generated sentences based on reasonableness and fluency, and both metrics are on a 1-3 Likert scale. The higher score indicates the model generates more reasonable and fluent endings. Fluency measures whether the generated sentences are coherent and fluent. Annotators should evaluate the grammar and the readability of the ending. Reasonableness measures whether the generated sentences are suitable for the story context. Annotators focus on whether the story ending is appropriate under the given story context. We randomly sample 200 generated results from CEG and GRF to conduct the human evaluation. The human evaluation results are summarized in Table 4.

**Table 4:** Human evaluation.

Model	Reasonableness	Fluency
GRF [7]	2.25	2.81
CEG (ours)	**2.35**	**2.84**

The numbers listed in the table are the score of metrics, which are 1-3 Likert scale. Higher reasonableness or fluency scores indicate that the results of the model are more reasonable and fluent.

The results show that our model performs better than GRF on reasonableness by 0.1 and on fluency by 0.03, which demonstrates our model’s capability of reasoning. GRF search for words that are just story keywords’ neighbors in ConceptNet. However, these words may not fit the story context, leading to lower reasonableness and fluency. In contrast, our model infers different causal events, which not only improve the reasonableness but also provide a sentence-level refinement to generate a more fluent story ending.

### Ablation study

In this part, we evaluate the influence of different attributes choices of relation in the generation model. We remove the different relation types in CEG and compare the results between the baseline and CEG. The results are summarized in Table 5.

**Table 5:** The effection of different relations.

Models	BLEU-1	BLEU-2
CEG w/o all	26.5	11.3
CEG w/o event	26.6	11.4
CEG w/o emotion	24.1	10.0
CEG w/o location	26.8	11.6
CEG w/o possession	26.5	11.1
CEG w/o other	26.6	11.4
CEG (ours)	**26.9**	**11.7**

The numbers listed in the table are percentages of BLEU-1 and BLEU-2. The higher BLEU scores, the better performance of the model. Baseline models are CEG models removing different relations. “w/o” means without and “w/o all” means without any relations. “w/o *r*” means without respective relation, where *r* represents relations defined GLUCOSE [9] including event, emotion, location, possession, and other attributes. For example, CEG w/o event is CEG model without event relation.

Comparing the results of different experiments, we find that all different relation types help the model perform better. CEG model infuses different types of causal events to comprehensively understand the story and generate a more reasonable ending based on the context and the BLEU scores decrease when CEG removes different relations. In addition, we find the BLEU-1 and BLEU-2 of CEG w/o emotion decline most among all ablation studies. We analyze the dataset and find that emotional events run through the main storyline. Therefore, it is important for the model to reason causal events about emotion to capture the story’s clues and then generate a reasonable ending. With the help of emotional knowledge, the model can more effectively grasp the main storyline and utilize knowledge of other types.

Furthermore, we conduct experiments to evaluate the effect of joint training. We use sentences of causal events as external knowledge instead of hidden states to enhance the story ending generation (CEG w/o joint). In this way, the loss of the generation model is detached from the inference model.

Table 6 shows that the performance of CEG w/o joint decreases by 1.9 on BLEU-1 and 1.7 on BLEU-2. Without joint training, the model has a gap between the causal events inference and story ending generation. The focus on causal events is scattered, disrupting the generation of models and leading to worse results. Joint training allows the model to focus on the events required to produce a reasonable story ending.

**Table 6:** The effect of joint training.

Models	BLEU-1	BLEU-2
CEG	**26.9**	**11.7**
CEG w/o joint	25.0	10.0

The numbers listed in the table are percentages of BLEU-1 and BLEU-2. The higher BLEU scores, the better performance of the model. “w/o joint” means without joint training.

### Case study

Table 7 provides examples of ROCStories and generated results of CEG and GRF. Combining endings generated by the two models with the story context, we find endings of GRF contain some logical errors. In the first case, the story context describes an accident during the trip to the beach, but GRF only captures vague development at the word level and ignores the casual information between the sentences. Meanwhile, CEG gets the inference that “Jen needs help” or “Jen wants safety” from the second sentence and “Jen is rescued” from the fourth sentence. Therefore, CEG can utilize the long-distance events and understand the story’s main idea, making the result more related to the context.

**Table 7:** Case study on the test set of ROCStorise.

Story Ending Generation	
**Story Context**	*Jen was at the beach with friends.*
	*She got knocked over by a strong wave.*
	*Jen was almost swept back out to sea.*
	*Friends saw what happened and went to help.*
**GRF**	Jen was okay but had a lot of fun.
**CEG**	Jen’s friends were able to save her life.
**Golden**	They were able to drag Jen back on shore.
**Story Context**	*Jill loves houses, so she decided to become a realtor.*
	*Jill signed up for a real estate licensing class.*
	*She attended all of the real estate classes.*
	*Jill studied very hard for the licensing exam.*
**GRF**	Jill now owns her dream house.
**CEG**	Jill passed the exam and now she is a realtor!
**Golden**	Jill took the real estate exam and passed it.

Furthermore, in the second case, GRF produces an ending that does not match the story context and ignores the story’s logic. In contrast to GRF, our model yields inference sentences “Jill feel(s) prepared” and “Jill possess(es) a real estate license”. We also show some inference sentences in Table 8. We find that the model has inferred many related events of the story context, which are useful for the generation.

**Table 8:** Case study on the test set of ROCStorise.

Story Ending Generation	
**Story Context**	*Jill loves houses, so she decided to become a realtor.*
	*Jill signed up for a real estate licensing class.*
	*She attended all of the real estate classes.*
	*Jill studied very hard for the licensing exam.*
**Inference Sentence**	*Jill passes the class*
	*Jill want(s) success*
	*Jill studied very hard for the exam*
	*Jill feel(s) prepared*
	*Jill passed the exam*
	*Jill possess(es) a real estate license*
**CEG**	Jill passed the exam and now she is a realtor!

## Conclusion

In this paper, we propose CEG, a joint model which infers the causal events behind the story to enhance the story ending generation. We first use the commonsense events inference model to convert GLUCOSE into a dynamic generative model to discover the commonsense events unseen in the original knowledge base. Then we propose a joint model trained on causal events inference task and story ending generation task. In the causal events inference task, the model reason causal events behind each story sentence by different relations to provide long-distance commonsense knowledge, which can help the model better understand the story and generate more reasonable story endings. The joint training and parameter sharing of the model can make causal events more related to the story ending. The experiments on ROCStories dataset show that our model outperforms the best baseline by 0.8 on BLEU-1 and 3.0 on DISTINCT-3, which indicates that our results are more commonsense and informative. Our model also performs better on reasonableness by 0.1 and on fluency by 0.03 in human evaluation, which shows that our model generates more reasonable and fluent endings. In addition, ablation studies demonstrate the effectiveness of commonsense events inference and joint training in our model.

Our model lacks interpretability in knowledge selection and has limited commonsense relationships. In the future, we plan to improve interpretability by explicitly selecting knowledge mechanisms. In addition, we will enhance reasoning ability by combining different commonsense knowledge bases, which can provide more extensive information and knowledge.
